# Patient Engagement With and Perspectives on a Mobile Health Home Spirometry Intervention: Mixed Methods Study

**DOI:** 10.2196/51236

**Published:** 2024-03-20

**Authors:** Andrew W Liu, William Brown, III, Ndubuisi E Madu, Ali R Maiorano, Olivia Bigazzi, Eli Medina, Christopher Sorric, Steven R Hays, Anobel Y Odisho

**Affiliations:** 1 Center for Digital Health Innovation University of California San Francisco, CA United States; 2 Department of Medicine University of California San Francisco, CA United States; 3 Department of Epidemiology and Biostatistics University of California San Francisco, CA United States; 4 Bakar Computational Health Sciences Institute University of California San Francisco, CA United States; 5 Department of Urology University of California San Francisco, CA United States

**Keywords:** mobile health, mHealth, remote patient monitoring, interview, interviews, dropout, attrition, eHealth, methods, telemedicine, statistics, numerical data, patient-centered care, spirometry, lung transplant, lung, transplant, transplants, transplantation, organ, organs, engagement, monitor, monitoring, pulmonary, respiratory, lungs, experience, experiences, device, devices, complication, complications

## Abstract

**Background:**

Patient engagement attrition in mobile health (mHealth) remote patient monitoring (RPM) programs decreases program benefits. Systemic disparities lead to inequities in RPM adoption and use. There is an urgent need to understand patients’ experiences with RPM in the real world, especially for patients who have stopped using the programs, as addressing issues faced by patients can increase the value of mHealth for patients and subsequently decrease attrition.

**Objective:**

This study sought to understand patient engagement and experiences in an RPM mHealth intervention in lung transplant recipients.

**Methods:**

Between May 4, 2020, and November 1, 2022, a total of 601 lung transplant recipients were enrolled in an mHealth RPM intervention to monitor lung function. The predictors of patient engagement were evaluated using multivariable logistic and linear regression. Semistructured interviews were conducted with 6 of 39 patients who had engaged in the first month but stopped using the program, and common themes were identified.

**Results:**

Patients who underwent transplant more than 1 year before enrollment in the program had 84% lower odds of engaging (odds ratio [OR] 0.16, 95% CI 0.07-0.35), 82% lower odds of submitting pulmonary function measurements (OR 0.18, 95% CI 0.09-0.33), and 78% lower odds of completing symptom checklists (OR 0.22, 95% CI 0.10-0.43). Patients whose primary language was not English had 78% lower odds of engaging compared to English speakers (OR 0.22, 95% CI 0.07-0.67). Interviews revealed 4 prominent themes: challenges with devices, communication breakdowns, a desire for more personal interactions and specific feedback with the care team about their results, understanding the purpose of the chat, and understanding how their data are used.

**Conclusions:**

Care delivery and patient experiences with RPM in lung transplant mHealth can be improved and made more equitable by tailoring outreach and enhancements toward non-English speakers and patients with a longer time between transplant and enrollment. Attention to designing programs to provide personalization through supplementary provider contact, education, and information transparency may decrease attrition rates.

## Introduction

Many large health systems have turned to remote patient monitoring (RPM) programs to improve population health outcomes, consistently engage with patients, and deliver care more efficiently at scale. RPM uses technology such as mobile devices (mHealth), wearables, or digital devices to communicate health information between patients and providers [1]. A 2022 systemic review found 268 published studies involving the use of RPM, reporting outcomes such as reduced health system costs, decreased hospitalizations, and improved patient quality of life [2,3]. The diversity of RPM clinical use cases includes dementia, diabetes, inflammatory bowel disease, sleep disorders, COVID-19, and cardiovascular disease, among many others [4-7].

However, RPM mHealth programs are most valuable when they maintain consistent patient engagement, allowing for adequate remote monitoring and increased data quality. High patient drop-off in RPM programs is common and seemingly inevitable upon implementation [8]. In a meta-analysis of 17 studies, 43% of patients stopped using the intervention before study completion [9]. In the same meta-analysis, observational real-world studies had even higher (49%) attrition compared to randomized controlled trials. Individual disengagement in RPM programs may arise from a wide range of factors, such as technological difficulties, lack of face-to-face encounters, or irrelevant content [10]. In addition, systemic disparities exist in terms of digital access and literacy, especially among vulnerable populations, leading to inequities in RPM adoption and use [10-13]. There is an urgent need to understand the patient experiences of RPM in real-world studies, especially for patients who have dropped off in engagement, as these patients may face untraditional and uncommunicated needs and challenges.

At the University of California, San Francisco (UCSF), a real-world RPM program for lung transplant recipients was launched in 2020 and is currently ongoing [14]. Patients used Bluetooth-enabled home spirometers to monitor pulmonary function and reported results and outcomes using a web-based chat interface. With the goal of engaging a higher proportion of patients and improving RPM compliance, we used a mixed methods approach to quantitatively identify the clinical and demographic predictors of patient engagement and conducted qualitative semistructured interviews with patients who had stopped using the home spirometry program after the first month to understand the nuances behind patient drop-off.

## Methods

### Home Spirometry Program

**Figure 1:**
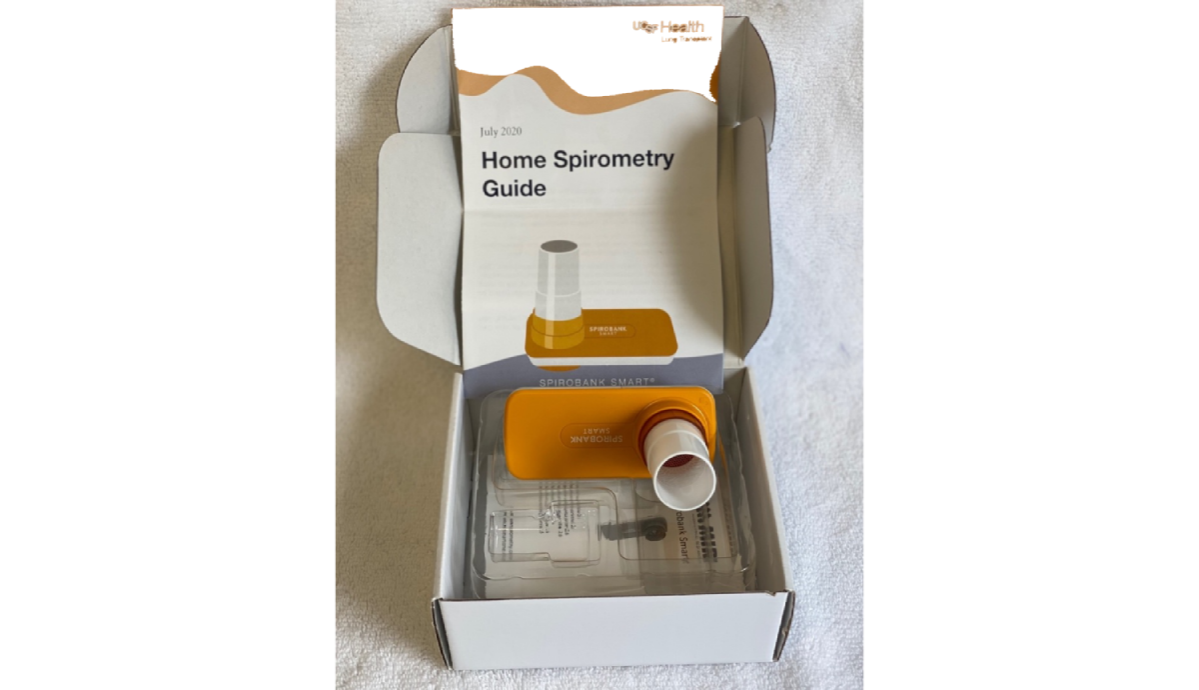
Home spirometry device and onboarding.

All patients who have had a lung transplant at UCSF are enrolled in an ongoing real-world RPM mHealth intervention that was implemented as a change in routine care [14]. The intervention is composed of an automated, English-only, chatbot-based symptom monitoring experience powered by a third-party vendor (Conversa Health, Inc), paired with a Bluetooth-enabled handheld home spirometer (Spirobank Smart or SmartOne, MIR Medical International Research) that allows patients to record their forced expiratory volume in the first second (FEV_1_) to assess and track their pulmonary function (Figure 1). The program was launched in May 2020, with all patients transplanted after May 1, 2020, automatically enrolled and given the chance to opt out. Additionally, all UCSF patients who had ever had a lung transplant were offered an opportunity to self-enroll (opt in) to the program at the time. In August 2020, all patients who had previously received a lung transplant and who had not self-enrolled were automatically bulk enrolled in the program, allowing them to opt out. Patients transplanted after May 2020 were onboarded in person during their posttransplant care, while patients transplanted before May 2020 were onboarded either digitally through mailed guides or during a routine outpatient follow-up visit.

**Figure 2:**
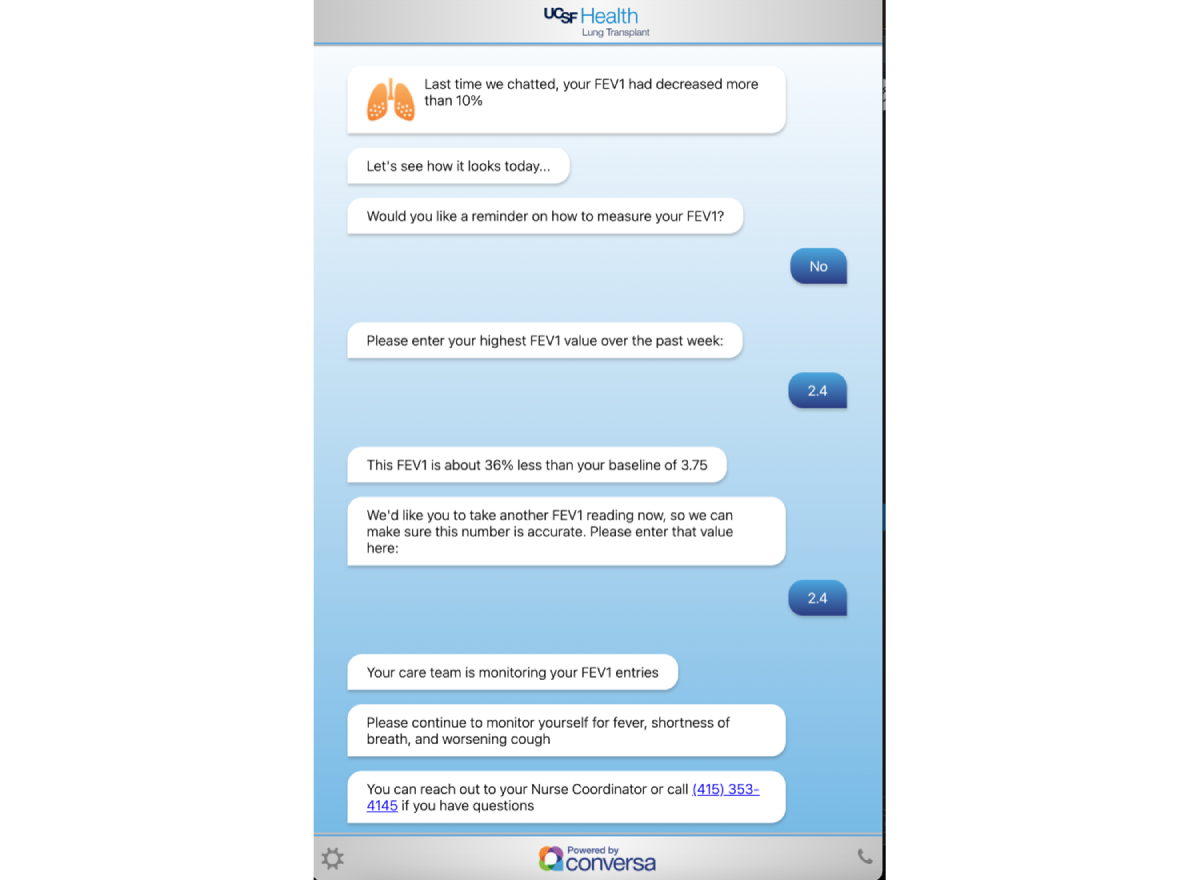
Screenshot of patient chat experience. FEV1: forced expiratory volume in the first second.

Through the automated chat, patients can complete individual modules, in which they can report symptoms, manually input new FEV_1_ measurements recorded from their Bluetooth spirometer, and receive educational content embedded into chat modules (Figure 2). The goal of the program is early detection of acute and chronic allograft dysfunction and infections. Any abnormal drops in pulmonary function (>10% from baseline for each patient) or concerning patient symptoms generated an immediate alert to an electronic health record shared in-basket that was monitored by the lung transplant care team. Patients were also provided with the clinic contact information and instructed to reach out for additional advice. The transplant team managed clinical findings at their discretion. Additionally, patients are expected to engage with routine automated chat prompts as part of their posttransplant care indefinitely to provide the most up-to-date spirometry and symptom data to their providers, who review with patients during outpatient follow-up visits. Initially, all patients were on a weekly chat reminder cadence, with a reversion to a daily reminder cadence if their condition deteriorated (as defined by a 10% drop in FEV_1_ or the reporting of concerning symptoms). In May 2021, the chat reminder cadence was changed to allow patients 1-year posttransplant with stable conditions to opt into a monthly cadence, with the possibility to return to a weekly or daily cadence if their condition deteriorated, and the eventual regraduation to weekly or monthly once they began to recover. Patients could self-initiate chat sessions at will.

### Predictors of Patient Engagement

The 3 primary outcomes were patient engagement with the intervention, including (1) module engagement, defined as completing at least 1 module of any chat, (2) spirometry engagement, defined as patients who submitted at least 1 home spirometry FEV_1_ value, or (3) symptom checklist engagement, defined as patients who responded to a symptom-reporting checklist at least once. Unengaged patients were defined as those who did not (1) complete any modules, (2) submit FEV_1_ values, or (3) complete symptom checklists. As submitting a FEV_1_ or a symptom checklist inherently resulted in the completion of a module, all patients classified as engaged by definitions 2 and 3 were also classified as engaged by definition 1, with definition 1 serving as the broadest outcome. It was possible for patients to have only engaged in educational modules without FEV_1_ or symptom submission, resulting in only meeting the module engagement definition without meeting the other 2. Additionally, as secondary outcomes, the number of times patients completed the chat, submitted FEV_1_ values, and symptom checklists were also calculated for each patient within the first year after enrollment. Since patients were asked to complete the chat at regularly scheduled repeated intervals, patients who died during their first year of enrollment were excluded from this secondary analysis.

Patient demographic and scheduling data were extracted from the electronic health record. This included patient age, transplant date, sex, race or ethnicity, address, insurance payor, primary language, and marital status. The primary diagnosis resulting in a need for transplantation was classified into 5 categories: restrictive disease, obstructive disease, cystic fibrosis or bronchiectasis, pulmonary hypertension, and other disease. Rural or urban status was assigned at the zip code level using the rural-urban community area codes classification [15]. Area Deprivation Index (ADI) national percentiles, based on a patient’s US census block group location, were used as a proxy measure of socioeconomic status [16].

Differences in the patient cohort conditioned on engagement status based on whether or not patients had completed any modules were compared using the chi-square test for categorical features and the 2-sample *t* test (2-tailed) for continuous features. Multivariate logistic and linear regression models were created to assess the predictors of engagement defined by chat module completion and longitudinal engagement, respectively. All analyses were performed using R (version 3.5.1; R Foundation for Statistical Computing), and a *P* value <.05 was considered significant.

### Qualitative Interviews

A convenience sample of patients (n=6) who initially engaged with the mHealth program in the first month but subsequently stopped using it (n=39) were recruited to participate in 40-minute semistructured qualitative interviews. The interviews were led by 1 of 2 user experience designers (NEM and ARM) and conducted through Zoom videoconferencing (Zoom Technologies Inc). Patients were invited to include their significant others or caretakers in the call to help provide additional context and reassurance. Patients were compensated with a US $25 gift card. Interviews were audio-recorded and transcribed. NEM, ARM, AWL, and OB then collaboratively synthesized results to determine concepts and themes by pairing quotes and notes to relevant themes. Three individuals who did not participate in the interviews (AWL, WB III, and AYO) reviewed the selected themes with accompanying quotes and selected quotes for presentation.

The qualitative interview opened with topics concerning the patient’s background, including transplant experience, current condition, the frequency of home spirometry, and chat use. Patients were prompted with screenshots of the chat experience and asked about their perceptions, program expectations, reasons for opting out and nonuse, and any challenges they experienced. Patients were also asked about whether solving the challenges they brought up would cause them to reengage at a higher rate. Finally, patients were also given the opportunity to ask questions and provide comments on anything not already touched upon.

### Ethical Considerations

The retrospective quantitative and prospective qualitative portions of this study, including patient compensation, were separately reviewed and approved by the University of California, San Francisco institutional review board (22-35950 and 22-35948).

## Results

### Overview

Between May 4, 2020, and November 1, 2022, a total of 626 patients were enrolled in the chat and 25 patients opted out, resulting in a total of 601 patients included in multivariate logistic regression analysis. Additionally, 33 patients died within their first year of enrollment, resulting in 568 patients included in multivariate linear regression modeling.

In total, 479 (79.7%) patients completed ≥1 module, 433 (72%) patients submitted ≥1 FEV_1_ value, and 438 (72.9%) patients submitted ≥1 symptom assessment. The engaged or unengaged cohorts when compared by module completion status were composed of patients of similar sex, race or ethnicity, rural or urban zip code status, marital status, primary language, insurance type, and diagnosis. Engaged patients were marginally younger (66.1 vs 65.2 years; *P*<.01), had more recent transplants (1.6 years since transplant for engaged patients vs 5.7 years for nonengaged patients; *P*<.01), and lived in areas with lower ADI national percentiles (14 vs 16; *P*<.01; Table 1).

**Table 1:** Patient demographics by module completion (engagement) status (n=601). Patients were considered engaged if they completed any chat modules, including the forced expiratory volume in the first second (FEV1)and symptom submission modules (definition 1).

			Not engaged participants (n=122)		Engaged participants (n=479)		*P* value	
Patient age (years), median (IQR)			66.1 (57.7-72.8)		65.2 (55.8-71.2)		<.01	
Tim since transplant (years), median (IQR)			5.7 (2.7-9.3)		1.6 (0.2-5.1)		<.01	
Transplant date ≥1 year of enrollment date, n (%)			109 (89.7)		279 (58.2)		<.01	
Submitted FEV_1_ data within 1 year, n (%)			—^a^		433 (90.4)			
Submitted symptom data within 1 year, n (%)			—		437 (91.4)			
FEV_1_ submissions in the first year, median (IQR)			—		26 (8-52)			
Symptom submissions in the first year, median (IQR)			—		24 (7-50)			
Total number of modules completed in the first year, median (IQR)			0 (0-0)		23 (9-40)			
Male, n (%)			76 (62.3)		260 (57.6)		.30	
**Ethnicity, n (%)**								.43
	Asian, Native Hawaiian, or other Pacific Islander	6 (5.3)		39 (8.5)				
	Black or African American	7 (6.1)		37 (8)				
	Hispanic or Latino	31 (27.2)		94 (20.4)				
	White	62 (54.4)		252 (54.7)				
	Other or unknown	8 (7)		39 (8.5)				
**Primary language, n (%)**								.13
	English	99 (86.8)		424 (92)				
	Other	15 (13.2)		37 (8)				
Urban, n (%)			114 (95)		439 (95.6)		.96	
ADI^b^ national percentile, median (IQR)			16 (8-28)		14 (5-30)		<.01	
**Marital status, n (%)**								.92
	Married or partnered	79 (69.3)		321 (69.6)				
	Single, separated, or other	35 (30.7)		140 (30.4)				
**Insurance, n (%)**								.09
	Commercial	20 (18.2)		127 (28)				
	Medicare	73 (66.4)		280 (61.8)				
	Medicaid	13 (11.8)		39 (8.6)				
	Other	4 (3.6)		7 (1.5)				
**Diagnosis, n (%)**								.77
	Restrictive disease	77 (72)		291 (72.4)				
	Obstructive disease	11 (13.1)		31 (12.4)				
	Cystic fibrosis	14 (10.3)		50 (7.7)				
	Pulmonary hypertension	3 (2.8)		21 (5.2)				
	Other disease	2 (1.9)		9 (2.2)				

^a^Not applicable.

^b^ADI: Area Deprivation Index.

### Predictors of Engagement

In a multivariate logistic regression model to identify predictors of engagement as defined by completion of a module (definition 1), patients who were enrolled ≥1 year from their transplant had 84% lower odds of engaging compared to those with more recent transplants (odds ratio [OR] 0.16, 95% CI 0.07-0.35; *P*<.01) when demographic factors (race or ethnicity, age, zip code status, insurance type, marital status, diagnosis, and socioeconomic status) were held constant. Similarly, patients with ≥1 year between transplant and enrollment had 82% lower odds of submitting pulmonary function measurements (OR 0.18, 95% CI 0.09-0.33; *P*<.01) and 78% lower odds of completing symptom checklists (OR 0.22, 95% CI 0.10-0.43; *P*<.01). Patients whose primary language was not English had 78% lower odds of engaging compared to primarily English speakers (OR 0.22, 95% CI 0.07-0.67; *P*<.01). Patient age, race or ethnicity, marital status, insurance type, sex, rural or urban zip code status, ADI national percentile ranking, and diagnosis were not found to have significant associations with engagement in the multivariate logistic regression model (Table 2).

In multivariate linear regression evaluating the number of modules completed in the first year of enrollment, single patient completed 8.69 fewer modules (95% CI –13.75 to –3.64; *P*<.01), patients with non-English primary languages completed 11.89 fewer modules (95% CI –20.62 to –3.17; *P*<.01), and patients with longer duration since transplant completed 15.22 fewer modules (95% CI –19.69 to –10.75; *P*<.01; Table 3). Multivariate analysis conducted on the number of FEV_1_ and symptom submissions within the first year found that longer duration between transplant and enrollment and having single or separated or other marital status remained predictors for longitudinal engagement (Table S1 in Multimedia Appendix 1).

**Table 2:** Predictors of the degree of engagement.^a^

Predictors			Number of FEV_1_ submissions					Number of symptom submissions		
			Estimate (95% CI)		*P* value		Estimate (95% CI)			*P* value
Age (years)			0.35 (–0.13 to 0.82)		.16		0.21 (–0.20 to 0.62)			.32
**Race or ethnicity (vs White)**										
	Asian, Native Hawaiian, or other Pacific Islander	0.90 (–13.53 to 7.62)		.92		–2.94 (–17.31 to 11.44)			.69	
	Black or African American	–8.32 (–0.13 to 0.82)		.23		–8.42 (–20.83 to 3.98)			.18	
	Hispanic or Latino	–2.96 (–21.82 to 5.19)		.58		–3.49 (–13.16 to 6.18)			.48	
	Other	5.91 (–6.73 to 18.56)		.36		5.60 (–6.23 to 17.42)			.35	
**Marital status (vs married or partnered)**										
	Single or separated or other	–13.86 (–22.13 to –5.59)		<.01		–11.59 (–19.23 to –3.95)			<.01	
**Insurance (vs commercial)**										
	Medicare	2.43 (–7.63 to 12.48)		.64		5.40 (–3.62 to 14.42)			.24	
	Medicaid	1.08 (–13.77 to 15.92)		.89		3.42 (–10.38 to 17.22)			.63	
	Other	–9.10 (–38.03 to 19.82)		.54		–11.27 (–34.86 to 12.31)			.35	
**Sex (vs male)**										
	Female	0.42 (–7.09 to 7.94)		.91		0.07 (–6.82 to 6.96)			.98	
**Primary language (vs English)**										
	Non-English	–11.38 (–25.65 to 2.90)		.12		–10.51 (–23.95 to 2.93)			.12	
Transplant date ≥1 year of enrollment date			–16.42 (–23.75 to –9.10)		<.01		–14.71 (–21.53 to –7.88)			.01
Rural			–1.55 (–19.04 to 15.94)		.86		0.88 (–15.77 to 17.54)			.92
ADI^b^ national percentile			–0.07 (–0.26 to 0.13)		.49		–0.09 (–0.27 to 0.09)			.32
**Diagnosis (vs restrictive disease)**										
	Cystic fibrosis	–3.15 (–19.65 to 13.36)		.71		–7.07 (–21.38 to 7.24)			.33	
	Obstructive disease	0.39 (–10.56 to 11.34)		.94		2.09 (–8.18 to 12.36)			.69	
	Pulmonary hypertension	–9.78 (–26.75 to 7.19)		.26		–8.49 (–24.10 to 7.12)			.29	
	Other disease	8.97 (–16.77 to 34.70)		.49		9.64 (–14.92 to 34.20)			.44	

^a^Results from a multivariate linear regression model (n=568).

^b^ADI: Area Deprivation Index.

**Table 3:** Predictors of module completion engagement. Results from a multivariate logistic regression model (n=601). Patients were considered engaged if they completed any chat modules, including the forced expiratory volume in 1 second and symptom submission modules (definition 1).

Predictors		OR^a^ (95% CI)	*P* value
Age (years)		0.99 (0.94-1.03)	.78
**Race or ethnicity (vs White)**			
	Asian, Native Hawaiian, or other Pacific Islander	1.94 (0.51-10.05)	.37
	Black or African American	1.19 (0.38-4.63)	.78
	Hispanic or Latino	1.20 (0.50-3.10)	.69
	Other	1.20 (0.44-3.88)	.74
**Marital status (vs married or partnered)**			
	Single or separated or other	0.95 (0.48-1.94)	.89
**Insurance (vs commercial)**			
	Medicare	0.78 (0.32-1.82)	.58
	Medicaid	0.38 (0.12-1.26)	.11
	Other	0.20 (0.03-1.28)	.08
**Sex (vs male)**			
	Female	1.25 (0.68-2.34)	.48
**Primary language (vs English)**			
	Non-English	0.22 (0.07-0.67)	<.01
Transplant date ≥1 year of enrollment date		0.16 (0.07-0.35)	<.01
Rural		0.53 (0.13-2.62)	.38
ADI^b^ national percentile		1.00 (0.98-1.02)	.95
**Diagnosis (vs restrictive disease)**			
	Cystic fibrosis	1.04 (0.28-4.43)	.96
	Obstructive disease	0.85 (0.35-2.23)	.74
	Pulmonary hypertension	0.75 (0.19-3.92)	.71
	Other disease	1.09 (0.15-22.20)	.94

^a^OR: odds ratio.

^b^ADI: Area Deprivation Index.

### Qualitative Interviews

#### Overview

Interviews were conducted with 2 male and 4 female patients, aged 35-70 years, and White and Latinx. Patients ranged from 1 to 18 years posttransplantation. One patient had his primary caretaker present as his proxy (Table 4). From the interviews, 4 key thematic concepts emerged: challenges with devices, communication breakdowns, desire for more personal interactions and specific feedback with the care team about data and concerns, and understanding the purpose of the care chat.

**Table 4:** Interviewed patient demographics (n=6). Patients were chosen based on having previously engaged in the first month but then subsequently having had no engagement afterward.

Patient	Age (years); sex	Time since transplant and final date of analysis^a^ (years)	Brief background
P1 and PR1	69; male	1.07	Latinx, primary language Spanish, and primary caretaker (PR1) present as proxy
P2	57; male	2.4	White, primary language English, and lives with a partner
P3	36; female	2.17	White and lives alone
P4	30; female	11.18	White and lives alone
P5	44; female	17.84	White and live with partner
P6	70; female	7.31	White and primary caretaker of the spouse

^a^November 1, 2022.

#### Patients Had Challenges With Devices

Patients reported a diverse set of difficulties associated with the use of their spirometer and mobile devices at home. Challenges encompassed all facets of the experience, such as difficulties with installing the spirometer app on their mobile device and connecting their spirometers through Bluetooth.

My machine will not connect to my phone or anybody else's phone. I tried unloading it, reloading it, even took it to AT&T because I'm not very tech savvy.

Participant 3

Low technological literacy was a barrier to engagement, and further product development is needed to make it easier to troubleshoot difficulties. One patient reported their preference to carry around an oximeter instead of using their spirometer due to a lack of confidence in their spirometer from experiencing device issues.

Upon use, patients also reported having issues with properly using their spirometer device. Changes in their position or actions led to inconsistent FEV_1_ measurements, which were perceived as unreliable in their eyes. For patients, receiving inconsistent FEV_1_ readings decreased their confidence, often triggering them to call their care team for guidance.

I have a hard time because I have to hold my phone, and I got to blow into it.... if I'm holding the phone down, now I'm having my neck down, it restricts some of my air.

Participant 2

Relatedly, patients reported frustration from using their home spirometer.

... it deflates me emotionally because it's always low.... I'm not hitting the 3. I'm hitting like, 1.9. So medically, I'm okay. But to me, it keeps on failing.

Participant 2

#### Communication Breakdowns

The transition for patients from using the automated care chat to reaching out to their care team via phone call or secure portal message led to communication breakdowns. Phone calls would occur back and forth between patients and different clinic staff before they were able to reach the right individual and receive relevant answers.

Sometimes if you have a question, I guess you have to call your nurse coordinator. And at times you have to wait for them to call you back.... So, I'm at work, so sometimes I miss their phone calls. Sometimes we play phone tag...

PR1

#### Patients Desire More Personal Interactions and Specific Feedback With the Care Team About Data and Concerns

Patients wanted additional opportunities to stay directly connected to their care team. They preferred to communicate with a member of their care team as opposed to entering their FEV_1_ or symptom measurements into the chat. “The chat doesn't really offer that opportunity to talk to someone in real life” [PR1].

The automated aspect of the care chat also resulted in a perceived technological barrier between patients and their care team. Patients were keenly aware of the automation and felt it did not provide enough incentive to stay engaged.

Lung transplant is a partnership and a lot of hands and a lot of faith and a lot of luck. And a lot of things came together to make a successful surgery.… You’re part of me now, you know. So yeah, any interaction is great. I’d rather talk to you. It's [the Care Chat] not very personal, very impersonal for the drastic surgery that we've had.

Participant 4

To patients, the automated chat responses did not provide enough contextual feedback after a FEV_1_ measurement was properly submitted to bring patients peace of mind. This resulted in concerns and a lack of confidence in at-home FEV_1_ measurements.

I don't know what I'm doing in the sense that I'm not getting any feedback from the other end. So,... thinking like, well, what am I doing ?]

Participant 2

Patients also wanted to know how their data were being used by the care team to assess their condition and wanted some acknowledgment that someone had reviewed their latest FEV_1_ measurement. The lack of feedback from their care team about their inputted values caused patients to want to disengage. There was a strong desire for additional patient-provider interaction around the FEV_1_ submission so that patients knew that their data were not going to be ignored.

I don't even know if the team ever looks at my what I submit via the care chat, because I don't ever get any feedback from them. So, it's kind of like, I think they see it, but I really don't know. And in that sense, it's like, well, do they really care? Maybe they do. Maybe they don't? This instantly makes me go, oh, well, if Dr. Hays is looking at these numbers, then I definitely want to provide them.

Participant 5

#### Patients Understand the Purpose of the Care Chat Experience

Patients strongly understood that their use of the care chat allowed providers to monitor their progress posttransplant. This resulted in patients being responsive to chat prompts. Furthermore, patients understood the clinical purpose of specifically collecting their FEV_1_ measurement.

I think the purpose is to monitor our lung function from a distance and for the doctors to be able to get more frequent measurements of our FEV1 without having us come in to do spirometry, or even go into our local hospital. It's kind of a way to keep tabs on us from home in a way that's safe for us and easier for them.

Participant 5

At its core, home spirometry helps capture a more comprehensive view of a patient’s well-being through consistent remote clinical data measurements and symptom reporting. This more comprehensive view not only allows their care team to be better informed but also respond faster to concerning changes in condition.

I think it's pretty great. I found it really helpful. And it's been nice for me to have like a reason to do my FEV1 and have a record of that. I like having more of a stand-up baseline that has more frequent measurements because I've always in clinic other than when I've been sick, it's been really stable, but it's nice to know at home like, oh yeah, this is really kind of where [my condition] lives.

Participant 5

## Discussion

### Principal Findings

We sought to understand engagement in a cohort of 601 patients who underwent lung transplant engaged in a real-world home spirometry mHealth intervention. Engagement and continued use of mHealth tools are critical to effective remote care. However, consistent, quality engagement in mHealth is a difficult challenge and often requires the tailoring of interventions to specific subpopulations [17,18]. We describe several novel predictors of engagement in this intervention and report major themes resulting from qualitative interviews. During our interviews, we found that patients who underwent lung transplant and who had engaged in mHealth RPM spirometry have a strong desire for improved connection to their care team and that when they feel disconnected, are unsure if their data are being received or reviewed, have trouble escalating their concerns to their care team, or experience device difficulties, they are more likely to stop using mobile health (mHealth) tools and home monitoring devices. As the first study (to our knowledge) to focus on in-depth lung transplant patient experiences with drop-off and RPM, this study brings to light patient perspectives that future interventions can learn from for program design specifics.

Multivariate modeling found that having a recent lung transplant (within 1 year of a patient’s enrollment date) was consistently found to have a positive association with engagement with the chatbot. One possibility of this is that patients with longer times since transplants might be more confident managing their care without the use of an mHealth app. However, as the potential for chronic allograft dysfunction remains high even 1-year posttransplant, it remains important to engage and reengage patients to reduce the risk of missed allograft dysfunction. Future outreach efforts toward long-term transplant patients to show them the benefits of participation will be required. Additionally, the factors serving as proxies for socioeconomic status (urban or rural status, ADI percentile, and health insurance type) were not found to be associated with engagement in the mHealth program while controlled for other factors during multivariate modeling. This may be due to the fact the spirometers and the chat were provided to patients at no charge. Other factors such as race or ethnicity and age were also not consistently found to be predictive under our 3 definitions of engagement. Finally, our multivariate analysis centered on examining binary engagement with the chat found that having a non-English primary language was associated with lower engagement. Previous literature has shown that patients with a non-English primary language are significantly less likely to engage in mHealth and telemedicine [18]. Our finding has led us to plan to translate the program into Spanish, which is our second largest language cohort (6% of patients), as a next step toward equity.

### Comparison With Previous Studies

Our study is not only the first to evaluate predictors of engagement of a mHealth RPM intervention in lung transplant recipients but also one of the first to examine RPM in a practice-wide, real-world setting, as opposed to a randomized controlled trial. Overall, the literature on factors influencing engagement in mHealth is mixed. Previous studies analyzing RPM programs have found age, income, and shorter time in program to be predictive of engagement [19]. Other studies have reported that drops in engagement are not associated with race or ethnicity, disease status, or geography [20]. Our analysis adds to the literature on RPM engagement: reporting that attrition over time remains a large complicated and multifaceted barrier and that trends in engagement are likely program and condition specific rather than being strongly influenced by socioeconomic factors. There is a need to continue to understand how social and clinical factors affect engagement in mHealth interventions to better engage vulnerable populations and not exacerbate existing disparities in care access. In addition, future work can be focused on comparative analyses between differences in engagement rates and outcome measurements (eg, emergency department visits, hospital readmissions).

In addition, interviews also revealed that some patients find the automated chatbot impersonal and crave additional interactions with their care teams. Program design can take this into account by creating defined instances and scenarios where providers should reach out in case of stress or confusion. Patients desire human contact during their posttransplant care; however, it remains a challenge to balance the need to remotely monitor a large cohort of patients in an automated fashion without overwhelming clinical teams with notifications and also providing a meaningful experience that patients value. Future developments can focus on determining chat design and flow improvements that will offer patients more individualization and education, fine-tuning automated triage mechanisms to identify the right time to have patient-provider interactions, and streamlining patient-provider communication channels to keep patients informed and engaged. Content creation can also be initiated with patient reassurance in mind, such as the development of educational videos featuring their providers or trusted sources. Finally, many patients struggled with proper device setup, spirometer technological difficulties, and confidently using the device to obtain consistent results, leading to confusion and a lack of trust in program quality. These results are consistent with findings showing that low technological literacy and confusion remain major barriers toward even more widespread adoption of RPM and mHealth [10,21,22]. Spirometry device satisfaction levels have been found to greatly influence patient engagement levels, as well as constant contact and follow-up by physicians to increase patient satisfaction [23,24]. In response, program enhancements were built to alleviate the cognitive burden on patients by streamlining onboarding and reducing the number of unnecessary chats. Future developments can aim to help patients build confidence and trust with home spirometry, by adding staff for more detailed onboarding, focusing on education for patients who have technological issues or concerns during the onboarding period, and chat design incorporating more person-to-person real-time feedback by providers.

### Limitations

Our study has several limitations. Our multivariate analysis did not account for health and digital literacy levels as these were not systematically assessed as part of routine clinical care and both have been shown to play major roles in a patient’s abilities to access health care. While our analysis factored in disease diagnosis type, there are likely additional clinical factors that may affect patient engagement, as transplant recipients with worse pulmonary function may be more apt to consistently engage to monitor their health. Future work can examine the efficacy of home spirometry monitoring in detecting adverse events when controlling for patients’ clinical conditions. Next, our study is a single-institution study of patients who have received lung transplant, and therefore the overall generalizability is reduced due to the specific nature of the patient’s condition, progression, and experiences with the program specifics such as Bluetooth spirometry. Interviewed patients were recruited only from those who initially dropped out after 1 month, and therefore the themes noted may only be representative of a dropped off in the engagement cohort. More work is required to elucidate the perspectives and reasons for continual engagement from patients whose engagements were consistent, steadily decreased over time, or dropped off at a later point in the program. In the future, we plan to interview patients who had excellent adherence to understand what factors are promoting adherence in these cases. Furthermore, the small sample size of our interview cohort increases the possibility of bias during the process of thematic analysis, particularly because we only interviewed 1 non-English speaker (using an interpreter, with the patient’s proxy present). Analyses from numerous implemented mHealth programs have shown that non-English speakers and patients of minority race or ethnicity are significantly less likely to engage in telemedicine [13,25,26]. Patient motivations for drop-off are nuanced and are likely strongly associated with sociocultural factors that can only be uncovered in larger, more diverse studies.

### Conclusions

An mHealth intervention consisting of home spirometry paired with an automated care chat results in high engagement rates in patients who have received a lung transplant, particularly in those with more recent transplants. Interviews conducted on patients who have dropped off in engagement revealed program challenges and areas where mHealth care delivery can be improved to reduce engagement attrition, including addressing technological barriers and improving patient confidence.

## Appendix

Multimedia Appendix 1 Predictors of the degree of engagement. Results from a multivariate logistic regression model.

## Abbreviations

ADI: Area Deprivation Index
FEV1: forced expiratory volume in the first second
mHealth: mobile health
OR: odds ratio
RPM: remote patient monitoring
UCSF: University of California, San Francisco
