# Supplementary material for: Patient Engagement With and Perspectives on a Mobile Health Home Spirometry Intervention: Mixed Methods Study
Source: JMIR Mhealth Uhealth. 2024 Mar 20;12:e51236. doi: 10.2196/51236 (PMC10993125; doi:10.2196/51236)
Supplement: Multimedia Appendix 1 [file mhealth_v12i1e51236_app1.docx]

**Table S1.** Predictors of degree of engagement (primary outcome of total number of completed chat modules). Results from a multivariate linear regression model (n=568).

|  | Estimate | 95% CI | *P* value |
| --- | --- | --- | --- |
| Age | 0.23 | –0.07 to 0.52 | .13 |
| Race or ethnicity (vs White) |  |  |  |
| Black or African American | –4.13 | –12.38 to 4.13 | .33 |
| Hispanic or Latino | 0.16 | –6.30 to 6.63 | .96 |
| Asian, Native Hawaiian, or Other Pacific Islander | 2.41 | –7.79 to 12.61 | .64 |
| Other | 3.25 | –4.48 to 10.98 | .41 |
| Marital status (vs married or partnered) |  |  |  |
| Single or separated or other | –8.63 | –13.69 to –3.58 | <.01 |
| Insurance (vs commercial) |  |  |  |
| Medicare | 1.49 | –4.66 – 7.63 | .63 |
| Medicaid | 2.00 | –7.08 to 11.07 | .67 |
| Other | –7.70 | –25.38 to 9.99 | .39 |
| Sex (vs Male) |  |  |  |
| Female | 0.07 | –4.53 to 4.66 | .98 |
| Primary Language (vs English) |  |  |  |
| Non-English | –11.91 | –20.64 – –3.18 | <0.01 |
| Transplant date ≥ 1 year of enrollment date | –15.34 | –19.82 to –10.86 | <0.01 |
| Rural | –4.42 | –15.11 to 6.28 | .42 |
| ADI national percentile | –0.04 | –0.16 – 0.08 | .50 |
| Diagnosis (vs restrictive disease) |  |  |  |
| Cystic fibrosis | –1.54 | –11.63 to 8.55 | .76 |
| Obstructive disease | 3.42 | –3.27 to 10.12 | .32 |
| Pulmonary hypertension | –8.58 | –18.95 to 1.80 | .10 |
| Other disease | –2.28 | –18.01 to 13.46 | .78 |
